# Vulvodynia: Pain Management Strategies

**DOI:** 10.3390/ph15121514

**Published:** 2022-12-05

**Authors:** Lucia Merlino, Luca Titi, Francesco Pugliese, Giulia D’Ovidio, Roberto Senatori, Carlo Della Rocca, Maria Grazia Piccioni

**Affiliations:** 1 Department of Maternal Infantile and Urological Sciences, Sapienza University of Rome, 00161 Rome, Italy; 2 Department of Anesthesia and Intensive Care Medicine, Sapienza University of Rome, 00161 Rome, Italy; 3 Italian Society of Colposcopy and Cervicovaginal Pathology (SICPV), 00186 Rome, Italy; 4 Department of Medical-Surgical Sciences and Biotechnologies, Sapienza University of Rome, 00161 Rome, Italy

**Keywords:** vulvodynia, vestibulodynia, pain, chronic pain, pain treatment

## Abstract

Background: Vulvodynia is defined in this international consensus as persistent vulvar pain that occurs for >3 months without an identifiable cause and with several potential associated factors. At present there is no univocal consensus in the therapeutic treatment of vulvodynia. The methods of intervention are based on various aspects including, above all, the management of painful symptoms. Methods: a research on scientific database such as “Pubmed”, “Medline Plus”, “Medscape” was conducted, using the words “women’s genital pain” and “vulvodynia” for the review of the scientific evidence on the assessment and treatment of women’s genital pain. Results: Among the drugs with pain-relieving action, the most effective in the treatment of vulvodynia would seem to be those with antidepressant and anticonvulsant action, even if their mechanisms of action are not known and there are still insufficient studies able to demonstrate their real validity. Among the least effective are non-steroidal anti-inflammatory drugs (NSAIDs) and corticosteroids. However, the ideal would seem to use a combined treatment with multiple types of drugs. Conclusions: Future studies are needed to draw up a unique therapeutic action plan that considers the stratification of patients with vulvodynia and the variability of the symptom.

## 1. Introduction

Vulvodynia is described as persistent vulvar pain lasting longer than three months where there is no recognisable organic cause of the disease, and with several potential associated factors [1]. This definition is derived from the 2015 international consensus of three scientific societies: the ISSVD (International Society for the Study of Vulvovaginal Disease), the ISSWSH (International Society for the Study of Women Sexual Health) and the IPPS (International Pelvic Pain Society). The aetiology is believed to depend on multiple factors, which will be described below. Therapeutic management consists of various types of options, including self-management, non-pharmacological therapies, pharmacological therapies and in some specific circumstances (vulvodynia unresponsive to medical therapy) even surgical therapies. Vulvodynia can lead to a drastic decrease in a patient’s overall quality of life, impacting in multiple aspects on the individual’s psycho-physical health and social relationships.

Vulvodynia as well as other co-morbidities that may affect the musculoskeletal system and the nervous system, those associated with pain such as fibromyalgia and irritable bowel syndrome, and psychosocial factors, are among the conditions referred to as psychosomatic [1]. Vulvodynia is a pathological condition that affects only the vulvar district but is enclosed in the “genito-pelvic syndromes” together with dyspareunia and vaginismus: the characteristics for the diagnostic distinction between these three syndromes are described in the Diagnostic and Statistical Manual of Mental Disorders, Fifth Edition (DSM-5), listed under the category of sexual dysfunction [2]. The 11th revision of the International Statistical Classification of Diseases and Related Health Problems came into force on 1 January 2022 and for the first time introduces a new classification for painful syndromes including vulvodynia. Symptoms primarily associated to vulvodynia include pain, which is the predominant symptom in affected patients, but also reduced sexual desire, arousal, sexual frequency and sexual satisfaction. This frequently leads to psychological alteration in the affected woman and her partner [3,4]. From a psychological point of view, feelings of discomfort, inadequacy towards the partner and low self-esteem are reported [5]. The great psychological impact that vulvodynia has on affected patients means that only 60 per cent of them go to the doctor, in 40 per cent of cases the disease will never be diagnosed [6].

## 2. Materials and Methods

A research on scientific database such as “Pubmed”, “Medline Plus”, “Medscape” was conducted for reviewing the scientific evidence on the assessment, diagnosis and treatment of female genital pain. We were primarily concerned with drug treatment options in these conditions.

Such items included vulvodynia, vestibulodynia, vulvar pain, vulvodynia treatment, vulvodynia drug treatments, introitail dyspareunia, vulvodinya associated comorbidities. It was included articles in English language and human trial literature with the aim of writing a narrative review of pharmacological options for pain management in vulvodynimic patients.

## 3. Results

### 3.1. Pathophysiology

Vulvodynia has traditionally been described as a disorder involving two spheres: on the one hand, physical alterations that cause the pain symptoms and, on the other, psychological and social problems that feed and perpetuate the pathological picture. In fact, according to the most recent accreditation, it is necessary to consider an integrated model that takes into account biopsychosocial factors and related impairments, identifying the organic and psychosocial mechanisms involved in the onset of syntoms, in the chronicity of the same and in the exacerbation of pain [7].

It is necessary to differentiate vulvodynia from pain associated with a specific vulvar disorder such as infectious causes (candidiasis, bacterial vaginosis), inflammatory forms (lichen sclerosus, contact dermatitis), neoplastic causes (Paget’s disease, squamous cell carcinoma) and neurological forms (pudendal nerve entrapment, spinal nerve compression). Vulvodynia proper can be subdivided into generalised and localised. Each of these two forms, in turn, can be:

- Provoked (sexual, non-sexual or both) “Provoked Vestibulodynia” (formerly Vulvar Vestibulitis Syndrome)
- Unprovoked: ‘Generalised Vulvodynia’.
- Mixed (provoked and unprovoked).

### 3.2. Pain Mechanisms

The neurophysiopathology of vulvodynia is varied and is characterised by sensory abnormalities of the peripheral and central nervous system [8,9]. Allodynia and hyperalgesia are typical features of vulvodynia and are often associated with other chronic pain conditions [9,10,11]. It would seem that the primum movens is the development of a chronic inflammatory framework of the vulvar mucosa that in the long run leads to an increase in the density of the nerve fibres of the vestibule [12,13]. This increase in local nerve fibre density appears to be mediated by the AT2 receptor in murine cultured cells, suggesting that a local renin-angiotensin inflammatory system could have an important role. The increased nerve fibre density in vulvodynia affected patients contrasts, however, with what genenrally occurs in patients with other peripheral pain neuropathies. Indeed, in these categories, there is often a reduction in intra-epidermal nerve fibre density [14]. Certainly further studies are needed to clarify the involvement of nerve fibres in the pathogenesis of this painful condition and perhaps help in the differentiation between vulvodynia and other peripheral pain neuropathies. Numerous psychophysical studies, that have investigated numerous sensory modalities- temperature, light touch, puncture or relative pressure on peripheral and central somatosensory channels- [15], have shown an increased pain sensitivity at the urogenital area in women with vulvodynia compared to healthy controls [16]. We know that women with vulvodynia experience an increased sensitivity to different extra-genital sensory stimuli [16,17], suggesting central sensitisation. This central sensitisation could explain the observed overlapping chronic pain conditions in this target of patients. A dysregulation of the pain modulation system is also suggested by functional data from resonance studies [16,17,18,19]. Moreover, there are structural resonance data in women with vulvodynia showed increased grey matter volume in the basal ganglia, sense-sorimotor cortices and hippocampus [19,20]; however other studies showed a reduction in grey matter volume in patients with chronic, total or regional pain [19]. This because of extreme variability of patients affected and because of the frequent early onset of clinical symptoms; patients with vulvodynia can suffer of other chronic pain syndrome [19]: it’s included in that set of central sensitivity syndromes, i.e., a group of heterogeneous syndromes including fibromyalgia, chronic fatigue syndrome, irritable bowel syndrome and temporomandibular joint disorder, characterised by painful symptoms such as pain and fatigue in the absence of clinically evident disease [19]. These syndromes are characterised by central sensory increases in neuroimaging studies, which could be a predisposing factor or a consequential effect. It is not possible to distinguish a ‘neuroimaging signature’ that defines any of these syndromes based on current literature [20].

*Inflammatory factors* Although several studies have obtained conflicting results, it is thought that there may be a correlation between the development of vulvodynia and a chronic inflammatory condition. Relevant findings shown a disproportion of mast cells in vestibular tissue with an higher number of these cells and a reduced systemic number of natural killer cells in vulvodynia affected women compared to controls [21]. In addition, there is evidence of recurrent infections with mycetes, especially Candida [22]; it has therefore been hypothesised that a deficiency in the number of natural killer cells may be correlated with recurrent Candida infections in these women. These data underline the importance of a detailed examination of the vaginal microflora in patients with vulvodynia.

*Neuroselective sensory dysfunction of the pudendal nerve* Murina et al. conducted a study to objectively assess vulvodynia using the current perception threshold (CPT) neurometer. In this study, neuroselective CPT measurements of the pudendal nerve were obtained at the perineum by a neurometer (Neurotron, Inc., Baltimore, MD, USA), using a constant alternating sinusoidal waveform electrical stimulus at frequencies of 2000, 250 and 5 Hz, in 20 healthy volunteers and 38 women with vulvodynia. The results of this study support neuroselective sensory dysfunction in generalised vulvodynia. The field is open for CPT measures in vulvodynia in the selection of therapeutic strategy, monitoring of treatment response and evaluation of vestibulodynia [23].

*Neuropathy of small autonomous nerve fibers* A condition characterised by intense pain sensations that usually begin in the extremities and may also include the sexual organs. Affected persons are unable to perceive pain when it is concentrated in very small areas. However, when subjected to normal stimuli, i.e., stimuli that in a healthy individual would not cause pain, the same individuals experience increased sensitivity to pain, hyperalgesia and painful sensations, a condition known as allodynia. The symptoms of small-fibre neuropathy can be varied, but pain is still the most common symptom, associated with burning, tingling, sudden painful sensations and loss of sensation [24].

Usually, diabetes or prediabetes are the underlying conditions that cause small fibre neuropathy. However, in some people, the cause of small fibre neuropathy may remain unknown, in which case the condition is called idiopathic small fibre neuropathy. Among various predisposing conditions, mutations in the SCN9A and SCN10A genes are often related to the development of small-fibre neuropathy. These genes code for sodium channels, which are necessary for cells to produce and transmit electrical impulses [25].

*Increased sensitivity of the peripheral regions of the body* Giesecke et al. conducted a study to assess both regional (vulvar) and general pain sensitivity in women with vulvodynia to determine whether both are increased, indicative of impaired central pain processing. Seventeen affected patients and 23 controls were included in the cross-sectional study. A newly developed vulvodolorimeter was used to assess vulvar pain. Peripheral pressure pain sensitivity was assessed by applying

(1) continuously increasing pressures at 3 bilateral positions (thumb, deltoid and shin),
(2) discrete pressure stimuli to the thumb using both an ascending and random sequence of variable pressures.

The results showed that pain resistance at all vulvar sites were lower in women with vulvodynia than in pain-free control subjects. Similarly, peripheral pain resistances were below the thumb in women with vulvodynia when obtained from discrete ascending or random scale paradigms, as well as at the thumb, deltoid and shin when tested with a dolorimeter. Results were similar in both those with generalised vulvar dysesthesia and those with localised vestibulodynia. The quantitative results obtained with the vulvodolorimeter and the more subjective cotton swab test routinely used in diagnosis were strongly correlated. Women with vulvodynia were thus shown to have significantly increased sensitivity to pressure pain in both the vulvar and peripheral regions of the body, suggesting a ‘central’ component in the mechanisms mediating this disorder. Both the new vulvodolorimeter and the thumb pressure stimulator may help in future experimental tests of this and related hypotheses [17].

*Impaired neuroadaptation in patients with longer pain duration* Neurophysiology studies have shown that repeated stimulation of a small area results in temporal changes in cortical activity, most notably a reduction in cortical response with extended stimulus duration. At the unicellular level, visual and somatosensory cortical pyramidal neurons show changes depending on the use of their receptive fields and response properties with repetitive stimulation. The adaptation regulated by the central nervous system depends on several factors (such as GABAergic and NMDA receptor-mediated neurotransmission, neuron-glia interactions) that regulate how cortical information is processed. It is possible that alterations at each of these regulatory levels may cause disturbances in the neuromodulation of affected subjects. Patients with vulvodynia were found to have increased sensitivity to sensory stimulation both in the genital regions and at sites distant from it. It is thought that not only a peripheral sensitisation is involved, but also a central abnormality, similar to what occurs in other patients with pain syndromes, implying a diffuse CNS disturbance [26]. The observation of increased tactile sensitivity of the skin area distant from the vulvar region, including the static thresholds of all subjects with vulvodynia in this report—is consistent with an altered central sensitisation that develops with chronic pain. The reason for this is that this type of feed-forward inhibition occurs in the signal arrival layer at the somatosensory cortical level [27], in which the inhibitory cells of the local layer directly receive thalamocortical input and, in turn, suppress the responses of the excitatory cells of the neighbouring layer to their thalamocortical drive, thereby refining its properties [28].

These inhibitory cells are more responsive to weak (near-threshold) afferent drive than excitatory layer cells, and thus, subthreshold or weak stimulus inputs will have the effect of increasing the threshold at which excitatory layer cells begin to respond to peripheral pre-stimuli. If this alteration is sensitive to the time dependence of the GABAb receptor, then the measurement itself could be an indicator that GABAb efficiency has been compromised in some individuals [29].

*Autonomic dysfunction* In several studies, it has been observed that vulvodynia is often accompanied by autonomic changes, although we are not yet able to determine whether this is the cause or a consequence. Among the most frequently reported autonomic changes are a higher resting pulse and lower blood pressure than in controls [2].

*Hormonal factors* The innervation of the female reproductive tract is regulated by gonadal hormone levels [30]. In women with vulvodynia, a reduction in pain symptoms has been observed in correlation with the ovulatory phase of the menstrual cycle (characterised by higher oestradiol values) and an increase in pain symptoms in the premenstrual phase (characterised by low oestrogen levels) [31].

*Muscle dysfunction* Vulvodynia is frequently associated with varying levels of pelvic floor muscle dysfunction, including increased tone and alterations in muscle contractility and control. Controlled studies using validated measurements such as 4D ultrasound or dynamometry have demonstrated alterations in pelvic floor muscles at rest, including hypertone, poor muscle control, hypersensitivity and impaired contractility [32,33]. Women may also show spontaneous and involuntary contraction of pelvic floor muscles during attempted vaginal penetration. Further evidence supporting a role of muscular dysfunction in vulvodynia is the frequent co-occurrence with fibromyalgia of the musculoskeletal disorder.

*Embryological factors* It has been supposed that factors influencing embryonic development may also play a role in the aetiopathogenesis of vulvodynia. In the embryo at the fifth week of gestation the cloaca is divided by the urorectal septum from which the perineum and the lateral tissue to the cloaca are formed; from the anterior tissue, the urogenital sinus is derive, from the posterior tissue, the anorectal canal is formed. From the urogenital sinus originates the vaginal vestibule in which urethra, vagina and greater vestibular glands open [34]. Several data suggest a significant recurrence of interstitial cystitis in women with vulvodynia hypothesizing an alteration of the urogenital endothelium but further studies would be needed to confirm this hypothesis.

*Genetic aspects* A genetic hypothesis on vulvodynia was advanced by analyzing familial women with PVD who underwent vestibulectomy [35]. Testifying to this is a certain trend observed in some families in the development of frequent vaginal infections, especially by Candida, which may facilitate the onset of vulvodynia [36,37], as well as an altered inflammatory response following hormonal changes due to the introduction of the oestrogen pill [38]. Among gene polymorphisms associated with dysregulation of nociception transmission, A118G levels in OPRM1 (coding for the μ-opioid receptor) and β-endorphins are associated with increased pain sensitivity indicating a possible gene predisposition related to endogenous pain modulation [39]. Other gene variants related to severe forms of vulvodynia include specific alleles of TRPV1, which encodes for the transient potential member of the cation channel receptor V 1 subfamily, and NGF, which encodes for nerve growth factor [40].

*Psychosocial factors* Psychosocial factors must be included among the possible causes of vulvodynia. It has been seen how sexual abuse can favour the development of this type of pathological condition, especially if it occurs in childhood, as well as periods of stress or inability to communicate with the partner.

*Other comorbidities* Finally, the study of comorbidities often associated with vuvlodynia contributes to a more complete understanding of the predisposing, precipitating and/or maintaining factors that contribute to vulvar pain. Vulvodynia is frequently associated with other somatic pain syndromes such as fibromyalgia, endometriosis, painful bladder syndrome and irritable bowel syndrome [41,42] but comorbidity with other pelvic disorders is poorly studied, under-diagnosed and under-treated. There is therefore a need for further epidemiological studies to improve the therapeutic efficacy of treatment of these conditions. Grazziottin et al. [43] in a cross-sectional study of data from the Vulvodynia Network (Vu-Net) project emphasised a close correlation between recurrent vulvar pain and a family history of diabetes mellitus, recurrent vulvovaginal candidiasis, urinary tract infections, irritable bowel syndrome, constipation, headaches, migraine and menstrual headaches, allergies, anxiety, dyschezia, disabling dysmenorrhoea/endometriosis and major depression [44].

## 4. Discussion

Actually, there is no general consensus on the treatment of vulvodynia, mainly due to the limited availability of randomized clinical trials with placebo controls [45]. However, it is possible to recognize and highlight various subgroups of vulvodynia, which allows us to make consistent use of the diagnostic and therapeutic modalities available to us. Regarding the therapeutic approach of vulvodynia, a step-by-step method of how to treat pelvic floor dysfunction and psychological and sexual health is recommended, along with medical management in various doses and combinations. Women with less severe forms of vulvodynia could benefit from adequate psychological support, an adeguate relationship/dialogue with their partner, and other assistance and support measures. In some cases, when patients have not responded to initial stage of treatment, a multidisciplinary team may be necessary, including gynecologists, physiotherapists, psychologists and/or sex therapists, dermatologists [46]. It is important to emphasize that the choice of treatment depends mainly on local availability for skilled sex therapy or cognitive behavioural therapy, pelvic floor physical therapy and medical or surgical management. Only some countries have easy access to all therapeutic possibilities.

In terms of specific treatments for vulvodynia, analgesic drugs are the best resource we have. The various drugs differ in their mode of action and administration.

Table 1 summarises all the main drug classes used in the treatment of vulvodynia.

Table 2 also shows the treatment strategy recommended by the Vulvodynia Nertwork study group. Interestingly, a multimodal intervention (consisting in the use of two or more types of therapy) was adopted in 74.3% of patients [45].

*Topical creams* Topical lidocaine with 2–5% gel or cream is often tried in women with vulvodynia to reduce nociceptive sensitivity of the skin and mucous membranes and for desensitization of vestibular nerves [47]. This can be applied overnight or even several times a day depending on the patient’s needs. In a double-blind RCT that compared topical lidocaine to placebo, no difference was found in pain response to the swab test in women with PVD [48]. Intermittent topical use of lidocaine may be useful for women with intense vestibular touch pain and may be used prior to vaginal penetration.

*Antidepressants* Tricyclic antidepressants (TCAs) are drugs considered first-line in the treatment of neuropathic pain [49]. The exact anti-analgesic mechanism of TCAs is not completely clear but it appears to be due to repeated β2-adrenergic stimulation increasing concentration of norepinephrine at the level of the synaptic cleft [46].

Amitriptyline is often used for generalised pain not necessarily associated with vulvodynia, however some efficacy has been observed when administered to patients reporting this painful condition [50]. The only RCT that evaluated TCAs in women with PVD was conducted on low-dose oral desipramine that, however, did not find a superior effect compared to placebo [51]. Other types of antidepressants used for neuropathic pain have not yet shown proven efficacy for vulvodynia.

*Anticonvulsants* Anticonvulsants are often used in the treatment of vulvar pain but have been evaluated in a very small number of PVD studies. Among the most used, we have gabapentine and cabergoline. Gabapentine is able to reduce the propagation of the painful signal by acting on voltage-dependent sodium channels and reducing the release of neurotransmitters at the presynaptic level. However, it was found that there are no differences in painful sensation by comparing two groups of women in which vulvar pain was evoked through rubbing with a vaginal swab on the vaginal fornixes, one group of which took gabapentine and the other of which is a control placebo group [52]. In another case control study, on the other hand, gabapentin was able to reduce painful sensation compared to placebo [53]. Further studies are certainly needed to verify the potential role of anticonvulsant drugs in the treatment of painful vulvar pathologies, especially considering that this category of drugs could be exploited in all those overlap syndromes, in generalized vulvodynia or even in the case of comorbidities.

*Anti-inflammatory therapies* At present, it is not yet known whether chronic inflammation can represent a fulcrum in the development or exacerbation of vulvodynia; however, as reported by affected patients, the use of oral or topical NSAIDs is not effective in improving the painful sensation. Similarly, attempts have been made to treat PVD with topical corticosteroids without satisfactory results [54].

*Future immuno-targeting therapies for vulvodynia* Studies are currently underway for drug targets that can influence different levels of the immune system. For example, it was found that in women with vulvodynia, there is an higher concentration of fibroblasts at the vestibular level that could trigger a very high toll-like receptor-mediated inflammatory response in comparison to a control group of women without vulvar/vaginal pain; it means that a disproportionate and uncontrolled innate immune response could be the cause of vulvodynia and could be considered an objective therapy in future research [55].

*Hormones* Another controversial topic is the ability of oestrogen-progestins to increase pain sensation in patients with vulvodynia. It was found that combining estrogen with testosterone and at the same time discontinuing the estrogen-progestogen pill, there was a reduction of vestibular pain in women with vulvodynia [56]. It seems that hormone replacement therapy should not be recommended in the treatment of painful vulvar diseases, although future studies may show that there is a subgroup of patients who can improve with the cessation of hormonal contraceptives in combination with hormonal topical treatment.

*Neurotoxic agents* The use of botulinum toxin A (BTA) to reduce hypertonicity and pelvic pain in women in whom PFPT has occurred does not improve symptom conception in patients who have tried this therapeutic strategy. However, the injection of BTA represents a simple and inexpensive outpatient procedure, furthermore favorable results have been reported with doses up to 100 U. Further randomized placebo-controlled studies on BTA are currently being carried out and it is hoped that the results obtained will help us to clarify which doses and above all which methods of injection are preferable and which subgroups of patients could benefit most from them.

*Combined approaches* As we have seen, there are different therapeutic and management possibilities for vulvodynia and pelvic pain but the effectiveness of all these options has been carefully studied only by applying a monotherapy; even if it is now clear that a multifarmacological and multimodal approach is certainly more effective. Indeed, often in clinical practice, there have been several positive findings among physicians and healthcare professionals using a wide variety of drugs, often in combination [57,58]. Many of these therapeutic approaches have not been studied in vivo in women with vulvodynia, but they may prove to be the best strategy. For example, the simultaneous use of neuroleptic drugs and antidepressant drugs could represent an optimal solution for all those over-lap conditions between vulvodynia and other mood disorders considering the high frequency with which these associations occur.

## 5. Conclusions

At present, the treatment of vulvodynia does not yet have a univocal consensus. Pharmacological therapy with analgesic drugs are widely used but are often not sufficient to counteract pain symptoms. It is often necessary to use combined strategies with the use of multiple drug classes associated with pelvic floor rehabilitation techniques and psychological support. Future studies are needed to draw up a unique therapeutic action plan that considers the stratification of patients with vulvodynia and the variability of the symptom.

## Figures and Tables

**Table 1:** This table describes the drugs used in the treatment of vulvodynia with the respective therapeutic regimens most frequent in the literature.

Drug Therapy	Regimen
**Anti-nociceptive agents**	
Lidocaine	Topical 5% ointment, every night for 7 weeks
	Topical 2% lidocaine gel, 5 times per day for 12 weeks
	5% cream, 4 times per day for 12 weeks
Tricyclic	Oral desipramine, administered daily, increasing dose from 25 mg to 150 mg (6 weeks) for 12 weeks
	Topical 2% amitriptyline cream, twice per day for 12 weeks
Serotonin-norepinephrine reuptake inhibitors	Oral milnacipran, 50–200 mg per day for 12 weeks
Capsaicin	Topical 0.025% cream, 20 min application (then removed) per day for 8 weeks
	Topical 0.05% cream, decreasing dose from twice per day to twice per week for 4 months
**Anti-convulsant agents**	
	2–6% topical cream, 8 weeks
Gabapentin	Highest tolerable oral dose between 1200 and 3000 mg per day for 8 weeks
	Oral gabapentin, 1200–3000 mg per day for 8 weeks
**Anti-inflammatory agents**	
	Submucosal methylprednisolone (1, 0.5, 0.3 mL) once per week for 3 weeks
Corticosteroids	0.05% Clobetasol propionate or 0.5% topical hydrocortisone ointment for 28 nights
	Topical 1% hydrocortisone cream, twice per day for 13 weeks
**Anti-neuroinflammatory agents**	
	200 mg 3 times daily for four months
Palmitoylethanolamide	400 mg/40 mg 2 times daily for two months
	400 mg/40 mg two times daily for three months
**Antioxidant agents**	
Alpha lipoic acid	300–600 mg tablets for at least 3 weeks (600 mg for the acute phase, 300 mg for the maintenance phase)
	1800 mg intravenously per week
**Neurotoxic agents**	
	20 U, single injection into the bulbocavernosus muscle
Botulinum toxin A	100 U, single injection into the bulbocavernosus muscle
	100 U, single injection into the levator ani muscle
	50 U (single injection), 100 U (single injection, repeated after 3 months)

**Table 2:** Recommended therapies for the treatment of vulvodynia by Vulvodynia Network Group [45].

Anticonvulsants (Neuromodulators)
**-First-line treatment**
-Amitriptyline oral: 1 drop = 2 mg; start with 26 mg and increase in steps of 5 mg, as tolerated, every 3–7 days up to 30 mg
**-Second-line treatment**
-Gabapentin (from 300 to 1500 mg/daily) or
-Pregabalin (from 50 to 150 mg/daily) or
-Duloxetine (from 30 to 60 mg/daily) * if the patient is among the non-responders, combined therapy is useful
**Neuronal anti-inflammatory drugs**
-Alpha-lipoic acid (600 mg/daily)
**Non-pharmacological treatments: rehabilitation therapy of the pelvic floor**
-Muscle rehabilitation exercises for the pelvic floor, such as internal stretching and hands-on massage, trigger point acupressure, external and internal tissue massage, electromyographic biofeedback
-Electrical nerve stimulation (TENS)
-Muscle relaxants: Diazepam, oral (2 mg to 10 mg/day) or Diazepam, vaginal 5 mg/day (off-label)
**Anti-fungal**
-Fluconazole 200 mg (3 times a day for 1 week then once a week for 4 weeks, then 1 tbl/15 days for 2 months, then 1 tbl/months for 3 months
-Itraconazole 100 mg (twice a day for 3 days, then 1 tbl/15 days for 3 months) * used in case of patient suffering from recurrent Candida infections

## Data Availability

Not applicable.

## References

[1] Bornstein J., Goldstein A.T., Stockdale C.K., Bergeron S., Pukall C., Zolnoun D., Coady D. (2016). 2015 ISSVD, ISSWSH and IPPS consensus terminology and classification of persistent vulvar pain and vulvodynia. Obstet. Gynecol..

[2] American Psychiatric Association (2013). Diagnostic and Statistical Manual of Mental Disorders.

[3] Arnold L.D., Bachmann G.A., Rosen R., Kelly S., Rhoads G.G. (2006). Vulvodynia: Characteristics and associations with comorbidities and quality of life. Obstet. Gynecol..

[4] Jodoin M., Bergeron S., Khalifé S., Dupuis M.-J., Desrochers G., Leclerc B. (2008). Male partners of women with provoked vestibulodynia: Attributions for pain and their implications for dyadic adjustment, sexual satisfaction, and psychological distress. J. Sex. Med..

[5] Desrochers G., Bergeron S., Landry T., Jodoin M. (2008). Do psychosexual factors play a role in the etiology of provoked vestibulodynia? A critical review. J. Sex. Marital Ther..

[6] Harlow B.L., Wise L.A., Stewart E.G. (2001). Prevalence and predictors of chronic lower genital tract discomfort. Am. J. Obstet. Gynecol..

[7] Bergeron S., Rosen N.O., Morin M. (2011). Genital pain in women: Beyond interference with intercourse. Pain.

[8] Pukall C.F., Goldstein A.T., Bergeron S., Foster D., Stein E., Kellogg-Spadt S., Bachmann G. (2016). Vulvodynia: Definition, prevalence, impact, and pathophysiological factors. J. Sex. Med..

[9] Wesselmann U., Bonham A.D., Foster D. (2014). Vulvodynia: Current state of the biological science. Pain.

[10] Reed B.D., Harlow S.D., Sen A., Edwards R.M., Chen D., Haefner H.K. (2012). Relationship between vulvodynia and chronic comorbid pain conditions. Obstet. Gynecol..

[11] Veasley C. Impact of Chronic Overlapping Pain Conditions on Public Health and the Urgent Need for Safe and Effective Treatment: 2015 Analysis and Policy Recommendations. Chronic Pain Research Alliance. www.chronicpainresearch.org/public/CPRA_WhitePaper_2015-FINAL-Digital.pdf.

[12] Bohm-Starke N., Hilliges M., Falconer C., Rylander E. (1998). Increased intraepithelial innervation in women with vulvar vestibulitis syndrome. Gynecol. Obstet. Investig..

[13] Tympanidis P., Casula M.A., Yiangou Y., Terenghi G., Dowd P., Anand P. (2004). Increased vanilloid receptor VR1 innervation in vulvodynia. Eur. J. Pain.

[14] Lauria G., Lombardi R. (2007). Skin biopsy: A new tool for diagnosing peripheral neuropathy. BMJ.

[15] Tominaga M., Takamori K. (2014). Itch and nerve fibers with special reference to atopic dermatitis: Therapeutic implications. J. Dermatol..

[16] Hansson P., Backonja M., Bouhassira D. (2007). Usefulness and limitations of quantitative sensory testing: Clinical and research application in neuropathic pain states. Pain.

[17] Giesecke J., Reed B.D., Haefner H.K., Giesecke T., Clauw D.J., Gracely R.H. (2004). Quantitative sensory testing in vulvodynia patients reveals increased peripheral pressure pain sensitivity. Obstet. Gynecol..

[18] Farmer M.A., Maykut C.A., Huberman J.S., Huang L., Khalifé S., Binik Y.M., Apkarian V.A., Schweinhardt P. (2013). Psychophysical properties of female genital sensation. Pain.

[19] Schweinhardt P., Kuchinad A., Pukall C., Bushnell M. (2008). Increased gray matter density in young women with chronic vulvar pain. Pain.

[20] Bhatt R.R., Gupta A., Rapkin A., Kilpatrick L.A., Hamadani K., Pazmany E., Van Oudenhove L., Stains J., Aerts L., Enzlin P. (2019). Altered gray matter volume in sensorimotor and thalamic regions associated with pain in localized provoked vulvodynia: A voxel-based morphometry study. Pain.

[21] Masterson B.J., Galask R.P., Ballas Z.K. (1996). Natural killer cell function in women with vestibulitis. J. Reprod. Med..

[22] Harlow B.L., Caron R.E., Parker S.E., Chatterjea D., Fox M.P., Nguyen R.H.N. (2017). Recurrent yeast infections and vulvodynia: Can we believe associations based on self-reported data?. J. Womens Health.

[23] Murina F., Bianco V., Radici G., Felice R., Signaroldi M. (2010). Electrodiagnostic functional sensory evaluation of patients with generalized vulvodynia: A pilot study. J. Low. Genit. Tract. Dis..

[24] Harris V., Fischer G., Bradford J.A. (2017). The aetiology of chronic vulval pain and entry dyspareunia: A retrospective review of 525 cases. Aust. N. Z. J. Obstet. Gynaecol..

[25] Graziottin A., Gambini D., Bertolasi L. (2015). Genital and sexual pain in women. Handb. Clin. Neurol..

[26] Pukall C.F., Strigo I.A., Binik Y.M., Amsel R., Khalifé S., Bushnell M.C. (2005). Neural correlates of painful genital touch in women with vulvar vestibulitis syndrome. Pain.

[27] Favorov O.V., Kursun O. (2011). Neocortical layer 4 as a pluripotent function linearizer. J. Neurophysiol..

[28] Cruikshank S.J., Lewis T.J., Connors B.W. (2007). Synaptic basis for intense thalamocortical activation of feedforward inhibitory cells in neocortex. Nat. Neurosci..

[29] Zhang Z., Zolnoun D.A., Francisco E.M., Holden J.K., Dennis R.G., Tommerdahl M. (2011). Altered central sensitization in subgroups of women with vulvodynia. Clin. J. Pain..

[30] Foster D.C., Dworkin R.H., Wood R.W. (2005). Effects of intradermal foot and forearm capsaicin injections in normal and vulvodynia-afflicted women. Pain.

[31] Ting A.Y., Blacklock A.D., Smith P.G. (2004). Estrogen regulates vaginal sensory and autonomic nerve density in the rat. Biol. Reprod..

[32] Wesselmann U., Garrett-Mayer E., Kaplan Gilpin A.M., Zhang L., Czakanski P.P. (2006). The influence of the ovarian cycle on mechanical hyperalgesia in vulvar vestibulitis–a neuropathic urogenital pain syndrome. Ann. Neurol..

[33] Morin M., Bergeron S., Khalife S., Mayrand M.-H., Binik Y.M. (2014). Morphometry of the pelvic floor muscles in women with and without provoked vestibulodynia using 4D ultrasound. J. Sex. Med..

[34] Morin M., Binik Y.M., Bourbonnais D., Khalifé S., Ouellet S., Bergeron S. (2017). Heightened pelvic floor muscle tone and altered contractility in women with provoked vestibulodynia. J. Sex. Med..

[35] Deliveliotou A., Creatsas G., Farage M.A., Maibach H.I. (2006). The Vulva: Anatomy, Physiology, and Pathology.

[36] Selo-Ojeme D.O., Paranjothy S., Onwude J.L. (2002). Interstitial cystitis coexisting with vulvar vestibulitis in a 4-year-old girl. Int. Urogynecol. J..

[37] Morgan T.K., Allen-Brady K.L., Monson M.A., Leclair C.M., Sharp H.T., Cannon-Albright L.A. (2016). Familiality analysis of provoked vestibulodynia treated by vestibulectomy supports genetic predisposition. Am. J. Obstet. Gynecol..

[38] Farmer M.A., Taylor A.M., Bailey A.L., Tuttle A.H., MacIntyre L.C., Milagrosa Z.E., Crissman H.P., Bennett G.J., Ribeiro-da-Silva A., Binik Y.M. (2011). Repeated vulvovaginal fungal infections cause persistent pain in a mouse model of vulvodynia. Sci. Transl. Med..

[39] Gerber S., Bongiovanni A.M., Ledger W.J., Witkin S.S. (2003). Interleukin-1β gene polymorphism in women with vulvar vestibulitis syndrome. Eur. J. Obstet. Gynecol. Reprod. Biol..

[40] Goldstein A.T., Belkin Z.R., Krapf J.M., Song W., Khera M., Jutrzonka S.L., Kim N.N., Burrows L.J., Goldstein I. (2014). Polymorphisms of the androgen receptor gene and hormonal contraceptive induced provoked vestibulodynia. J. Sex. Med..

[41] Heddini U., Johannesson U., Grönbladh A., Nyberg F., Nilsson K.W., Bohm-Starke N. (2014). A118G polymorphism in the μ-opioid receptor gene and levels of β-endorphin are associated with provoked vestibulodynia and pressure pain sensitivity. Scand. J. Pain.

[42] Kalfon L., Azran A., Farajun Y., Golan-Hamu O., Toben A., Abramov L., Yeshaya A., Yakir O., Zarfati D., Falik Zaccai T.C. (2019). Localized provoked vulvodynia: Association with nerve growth factor and transient receptor potential vanilloid type 1 genes polymorphisms. J. Low. Genit. Tract Dis..

[43] Falsetta M.L., Foster D.C., Bonham A.D., Phipps R.P. (2017). A review of the available clinical therapies for vulvodynia management and new data implicating proinflammatory mediators in pain elicitation. BJOG.

[44] Backman H., Widenbrant M., Bohm-Starke N., Dahlof L.G. (2008). Combined physical and psychosexual therapy for provoked vestibulodynia-an evaluation of a multidisciplinary treatment model. J. Sex Res..

[45] LePage K., Selk A. (2016). What Do Patients Want? A Needs Assessment of Vulvodynia Patients Attending a Vulvar Diseases Clinic. Sex Med..

[46] Zolnoun D.A., Hartmann K.E., Steege J.F. (2003). Overnight 5% lidocaine ointment for treatment of vulvar vestibulitis. Obstet. Gynecol..

[47] Foster D.C., Kotok M.B., Huang L., Watts A., Oakes D., Howard F.M., Poleshuck E.L., Stodgell C.J., Dworkin R.H. (2010). Oral desipramine and topical lidocaine for vulvodynia: A randomized controlled trial. Obstet. Gynecol..

[48] Holbech J.V., Jung A., Jonsson T., Wanning M., Bredahl C., Bach F.W. (2017). Combination treatment of neuropathic pain: Danish expert recommendations based on a Delphi process. J. Pain Res..

[49] Leo R.J. (2013). A systematic review of the utility of anticonvulsant pharmacotherapy in the treatment of vulvodynia pain. J. Sex. Med..

[50] Brown C.S., Bachmann G.A., Wan J., Foster D.C., Gabapentin (GABA) Study Group (2018). Gabapentin for the treatment of vulvodynia: A randomized controlled trial. Obstet. Gynecol..

[51] Bachmann G.A., Brown C.S., Phillips N.A., Rawlinson L.A., Yu X., Wood R., Foster D.C. (2019). Effect of gabapentin on sexual function in vulvodynia: A randomized, placebo-controlled trial. Am. J. Obstet. Gynecol..

[52] Bergeron S., Khalifé S., Dupuis M.-J., McDuff P. (2016). A randomized clinical trial comparing group cognitivebehavioral therapy and a topical steroid for women with dyspareunia. J. Consult. Clin. Psychol..

[53] Falsetta M.L., Foster D.C., Woeller C.F., Pollock S.J., Bonham A.D., Piekna-Przybylska D., Maggirwar S.B., Haidaris C.G., Phipps R.P. (2018). Toll-like receptor signaling contributes to proinflammatory mediator production in localized provoked vulvodynia. J. Low. Genit. Tract Dis..

[54] Burrows L.J., Goldstein A.T. (2013). The treatment of vestibulodynia with topical estradiol and testosterone. Sex. Med..

[55] Reed B.D., Haefner H., Edwards L. (2008). A survey on diagnosis and treatment of vulvodynia among vulvodynia researchers and members of the International Society for the Study of Vulvovaginal Diseases. J. Reprod. Med..

[56] Lua L.L., Hollette Y., Parm P., Allenback G., Dandolu V. (2017). Current practice patterns for management of vulvodynia in the United States. Arch. Gynecol. Obstet..

[57] Brotto L.A., Yong P., Smith K.B., Sadownik L.A. (2015). Impact of a multidisciplinary vulvodynia program on sexual functioning and dyspareunia. J. Sex Med..

[58] Connor J.J., Haviland M., Brady S.S., Robinson B.B.E., Harlow B.L. (2020). Psychosocial Factors Influence Sexual Satisfaction among Women with Vulvodynia. J. Sex Marital Ther..

